# 

**Published:** 1898-11-12

**Authors:** 


					tHe HOSPITAL. ?< The standard of highest purity."?Lancet, Nov. 12th, 1898.
CADBURY'S ft* fJT CADBURY'S
COOOA Jnl JE2_ COCOA
tBSOLUTkLY PURE. NO DRU{*S OR ALKALIES USED
vsaarr W?s turn Lhfibluabx
?0. 633. Vol. XXY. LSE5S?] Saturday,
Nov. 12th, 1898.
^Snb.cnpu? ' ermB.J palCE TWOPENCE.

				

